# Increased Plasma Levels of Danger-Associated Molecular Patterns Are Associated With Immune Suppression and Postoperative Infections in Patients Undergoing Cytoreductive Surgery and Hyperthermic Intraperitoneal Chemotherapy

**DOI:** 10.3389/fimmu.2018.00663

**Published:** 2018-04-05

**Authors:** Guus P. Leijte, Hettie Custers, Jelle Gerretsen, Amon Heijne, Johannes Roth, Thomas Vogl, Gert J. Scheffer, Peter Pickkers, Matthijs Kox

**Affiliations:** ^1^Department of Intensive Care Medicine, Radboud University Medical Center, Nijmegen, Netherlands; ^2^Radboud Center for Infectious Diseases, Radboud University Medical Center, Nijmegen, Netherlands; ^3^Department of Anesthesiology, Radboud University Medical Center, Nijmegen, Netherlands; ^4^Institute of Immunology, University of Münster, Münster, Germany

**Keywords:** danger-associated molecular patterns, high mobility group box 1, immune suppression, HLA-DR, postoperative infections, cytoreductive surgery, hyperthermic intraperitoneal chemotherapy

## Abstract

**Introduction:**

Danger-associated molecular patterns (DAMPs) can elicit immune responses and may subsequently induce an immune-suppressed state. Previous work showed that increased plasma levels of DAMPs are associated with immune suppression and increased susceptibility toward infections in trauma patients. Like trauma, major surgical procedures, such as cytoreductive surgery (CRS) combined with hyperthermic intraperitoneal chemotherapy (HIPEC), are also thought to cause profound DAMP release. Furthermore, the incidence of postoperative infections in these patients, ranging from 10 to 36%, is very high compared to that observed in patients undergoing other major surgical procedures. We hypothesized that the double hit of surgical trauma (CRS) in combination with HIPEC causes excessive DAMP release, which in turn contributes to the development of immune suppression. To investigate this, we assessed DAMP release in patients undergoing CRS-HIPEC, and investigated its relationship with immune suppression and postoperative infections.

**Methods:**

In 20 patients undergoing CRS-HIPEC, blood was obtained at five time points: just before surgery (baseline), after CRS, after HIPEC, at ICU admission, and 1 day after surgery. Circulating levels of DAMPs [heat shock protein (HSP)70, high mobility group box (HMGB)1, S100A12, S100A8/S100A9, nuclear (n)DNA, mitochondrial (mt)DNA, lactate dehydrogenase (LDH), a marker of unscheduled cell death], and cytokines [tumor necrosis factor (TNF)α, IL-6, IL-8, IL-10, macrophage inflammatory protein (MIP)-1α, MIP-1β, and MCP-1] were measured. The extent of immune suppression was determined by measuring HLA-DR gene expression and *ex vivo* leukocytic cytokine production capacity.

**Results:**

Plasma levels of DAMPs (maximum fold increases of HSP70: 2.1 [1.5–2.8], HMGB1: 5.9 [3.2–9.8], S100A8/S100A9: 3.6 [1.8–5.6], S100A12: 2.6 [1.8–4.3], nDNA 3.9 [1.0–10.8], LDH 1.7 [1.2–2.5]), and all measured cytokines increased profoundly following CRS-HIPEC. Evidence of immune suppression was already apparent during the procedure, illustrated by a decrease of HLA-DR expression compared with baseline (0.5-fold [0.3–0.9]) and diminished *ex vivo* pro-inflammatory cytokine production capacity. The increase in HMGB1 levels correlated with the decrease in HLA-DR expression (*r* = −0.46, *p* = 0.04), and peak HMGB1 concentrations were significantly higher in the five patients who went on to develop a postoperative infection (p = 0.04).

**Conclusion:**

CRS-HIPEC is associated with profound DAMP release and immune suppression, and plasma HMGB1 levels are related with the occurrence of postoperative infections in these patients.

## Introduction

Peritoneal carcinomatosis (PC), as a result of dissemination of gastrointestinal or gynecological cancer into the peritoneal cavity, was formerly regarded as an uncurable condition. However, recent advances in our understanding of the pathophysiology of tumor spread indicate that PC is a loco-regional disease admissible for curative treatment (1). Cytoreductive surgery (CRS) combined with hyperthermic intraperitoneal chemotherapy (HIPEC) is now advocated as an effective strategy in patients with the peritoneum as the only site of dissemination (2). In this selected group of patients, CRS-HIPEC results in improved survival for both colorectal (3–5) and ovarian malignancies (6, 7) when compared to conventional chemotherapy. Nonetheless, CRS-HIPEC remains a complex and challenging procedure, associated with postoperative hemodynamic instability, coagulopathy, and metabolic alterations (8, 9). Therefore, the observed survival benefits come at the cost of high postoperative morbidity, with reported treatment-related complication rates of 23–44% for colorectal (5), and 31% for ovarian cancer (7). The most prevalent complication is postoperative infection, and strikingly, its incidence (10–36%) is very high compared with that observed in patients undergoing other major (abdominal) surgical procedures (8, 10–12).

In a large cohort of trauma patients, we have recently demonstrated that release of danger-associated molecular patterns (DAMPs) is associated with the development of immunosuppression and increased susceptibility toward nosocomial infections (13). When DAMPs are released upon cellular stress/damage they can bind to pattern recognition receptors on antigen presenting cells (14), and thereby elicit pro-inflammatory responses and a subsequent state of immune suppression (15). This DAMP-induced immunosuppression captures many hallmarks of sepsis-induced immunosuppression, and may increase susceptibility toward infections (16, 17).

Like trauma, major surgical procedures and chemotherapy are thought to cause profound cellular stress and consequent DAMP release (18, 19). This led us to hypothesize that the CRS-HIPEC procedure, comprising a double hit of surgical trauma and hyperthermic chemotherapy, causes excessive DAMP release, which in turn contributes to a systemic inflammatory response and the development of immune suppression (20). This would provide an explanation for the observed high susceptibility toward postoperative infections. The aim of this study was to investigate DAMP release in patients undergoing CRS-HIPEC and elucidate its relationship with the development of immune suppression and postoperative infections.

## Materials and Methods

### Study Population

Between April 2016 and September 2017, 20 adult patients undergoing a CRS-HIPEC procedure at the Radboud University Medical Center (Radboudumc) were included in this prospective observational study. Patients were diagnosed with peritoneal metastasis due to a primary colorectal or ovarian malignancy. Exclusion criteria were the use of steroids (all dosages) or other immunomodulatory medication (e.g., after organ transplant) prior to or during the study period. The study was carried out in accordance with the recommendations and approved by the local ethical committee (CMO Arnhem-Nijmegen; no. 2017-3607) with written informed consent from all subjects. Control samples were obtained from healthy male volunteers (median age 22 [21–24] years) participating in two human endotoxemia studies (12 subjects participating in NCT01835457 (21) and 30 subjects participating in NCT02922673), which were also approved by the local ethics committee (CMO Arnhem-Nijmegen; no’s. 2012-455 and 2016-2550). All healthy volunteers provided written informed consent and samples were obtained prior to any interventions. All study procedures were conducted in accordance with the declaration of Helsinki, including current revisions, and Good Clinical Practice guidelines.

### CRS-HIPEC Procedure

All patients underwent a CRS-HIPEC procedure at the Radboudumc according to the Radboudumc protocols, and performed by an experienced team. The anesthesia protocol was standardized and consisted of general anesthesia in combination with an epidural catheter. All patients received antibiotic prophylaxis (ceftriaxon 2 g i.v. on the evening before surgery and metronidazol 500 mg i.v. 60 min before surgery, of which the latter was repeated when the total length of surgery exceeded 8 h). After complete CRS, all subjects underwent a HIPEC procedure, consisting of intraperitoneal circulation of oxaliplatin (total dose of 460 mg/m^2^ in dextrose 5% solution during 30 min, 42–43°C) preceded by intravenous administration of 5-fluorouracil (total dose 400 mg/m^2^ in 1 h) and leucovorin (total dose 20 mg/m^2^ in 30 min). Following surgery, all patients were admitted to the intensive care unit (ICU). Postoperative analgesia comprised a combination of S-ketamine i.v. (0–10 mg/h) and Patient-Controlled Epidural Anesthesia (PCEA, with ropivacaine and/or sufentanil (max 12 ml/h) for up to 5 days). To prevent postoperative nausea and vomiting, patients received ondansetron (3 times daily 4 mg) and metoclopramide (3 times daily 10 mg if needed). During ICU stay, selective digestive tract decontamination was administered to all patients as part of the standard Radboudumc ICU protocol (22). None of the patients was treated with anti-inflammatory drugs or steroids during the study period.

### Sample Collection

Blood was obtained from the arterial cannula at five time points; directly after anesthetic induction (baseline), after CRS, after the HIPEC procedure, at ICU admission, and 24 h after start of surgery. Ethylenediaminetetraacetic acid (EDTA) anticoagulated blood was centrifuged immediately after withdrawal at 1,600 × *g* at 4°C for 10 min, after which plasma was stored at −80°C until analysis of heat shock protein (HSP)70, S100A8/S100A9, S100A12, and cytokine levels. To determine plasma levels of high mobility group box (HMGB)1, lactate dehydrogenase (LDH), nuclear (n)DNA, and mitochondrial (mt)DNA, EDTA plasma was centrifuged again at 16,000 × *g* at 4°C for 10 min to remove potential remaining platelets and cell debris, after which it was stored at −80°C until further analysis. The extent of immune suppression was determined by measuring cytokine production capacity of leukocytes *ex vivo* stimulated with lipopolysaccharide (LPS), and leukocytic HLA-DRA mRNA expression. Blood for *ex vivo* stimulation was collected in lithium heparin (LH) tubes, and blood for mRNA analysis was sampled in PAXgene blood RNA tubes (Qiagen, Valencia, CA, USA) and stored according to the manufacturer’s instructions. Furthermore, demographic data and clinical parameters were obtained from electronic patient files. Postoperative infections were scored by two independent physicians and classified as severe when organ dysfunction was present.

### Plasma DAMP Levels

We measured DAMPs reflecting cellular stress (23), cellular decay (24), and hyperthermic stress (25). The plasma concentrations of HSP70/HSPA1A and S100A12 (ENRAGE) were determined batchwise using enzyme-linked immunosorbent assays (ELISA) according to the manufacturer’s instructions (R&D systems, Minneapolis, MN, USA). Plasma levels of HMGB1 were determined using the Shino-test ELISA Kit according to the manufacturer’s instructions (IBL International GmbH, Hamburg, Germany). Concentrations of S100A8/S100A9 (MRP8/MRP14, calprotectin) in plasma were determined using a sandwich ELISA at the Institute of Immunology, Muenster, Germany, as described previously (26). For nDNA and mtDNA measurements, detailed methodology can be found elsewhere (13). Briefly, DNA was isolated using the QIAamp DNA Blood Midi Kit (Qiagen, Valencia, CA, USA) and qPCR was performed using iQ SYBR Green PCR Master Mix (Bio-Rad Laboratories, Hercules, CA, USA) on a CFX96 Real-Time PCR Detection System (Bio-Rad Laboratories, Hercules, CA, USA). For nDNA detection, primers for GAPDH were used: forward 5′-AGCACCCCTGGCCAAGGTCA-3′ and reverse 5-CGGCAGGGAGGAGCCAGTCT-3′. For mtDNA detection, primers for MT-ND1 were used: forward 5′-GCCCCAACGTTGTAGGCCCC-3′ and reverse 5′AGCTAAGGTCGGGGCGGTGA-3′. Patient DNA was compared to DNA isolated from whole blood obtained from healthy volunteers. Plasma levels of nDNA and mtDNA are expressed as fold change relative to the mean Ct value in healthy volunteers using the formula 2^ΔCt^. As a general marker of unscheduled cell death, plasma levels of LDH were determined by the Laboratory of Clinical Chemistry of Radboudumc (Cobas C8000, Roche Diagnostics, Indianapolis, IN, USA).

### Plasma Cytokine Concentrations

Concentrations of tumor necrosis factor (TNF)α, interleukin (IL)-6, IL-8, IL-10, macrophage inflammatory protein (MIP)-1α, MIP-1β, and monocyte chemoattractant protein (MCP)-1 were determined batchwise using a simultaneous luminex assay (Milliplex, Millipore, Billerica, MA, USA) according to the manufacturer’s instructions.

### *Ex Vivo* Cytokine Production

Leukocyte cytokine production capacity was determined by challenging 0.5 mL LH-anticoagulated whole blood with 10 ng/mL LPS *ex vivo* at 37°C for 24 h using an in-house developed system with prefilled tubes described in detail elsewhere (27). Concentrations of TNFα, IL-6, and IL-10 in supernatants of stimulated cultures were determined batchwise using ELISA’s according to the manufacturer’s instructions (R&D systems, Minneapolis, MN, USA) and corrected for blood monocyte count determined by the Laboratory of Clinical Chemistry of the Radboudumc (Sysmex XE-5000, Sysmex Nederland B.V., Etten-Leur, The Netherlands).

### HLA-DR Expression

HLA-DR expression was determined as previously reported (13). Briefly, RNA was isolated using the Paxgene Blood RNA kit (Qiagen, Valencia, CA, USA) and transcribed into cDNA using the iScript cDNA Synthesis kit (Bio-rad, Hercules, CA, USA). qPCR analysis was performed using TaqMan gene expression assays (Life Technologies, Paisley, UK) for the reference gene peptidylpropylisomerase B (PPIB) (#Hs00168719_m1) and HLA-DRA (#Hs00219575_m1) on a CFX96 Real-Time PCR Detection System (Bio-Rad, Hercules, CA, USA). The HLA-DRA gene was used because it was shown to correlate well with flow cytometric analysis of monocytic HLA-DR (28), an established marker of immune suppression. HLA-DRA expression levels are expressed as fold change relative to the expression of PPIB in the same sample using the formula 2^ΔCt^.

### Statistical Analysis

All data were not normally distributed (according to the Shapiro–Wilk test) and are, therefore, presented as median [interquartile range]. Differences between baseline patient data and healthy controls, and between patients with and without postoperative infections were analyzed using Mann–Whitney *U*-tests, and differences over time within patients were analyzed using Friedman tests with Dunn’s *post hoc* tests. The correlations between different DAMPs as well as between DAMPs and cytokines were calculated using the method for repeated observations described by Bland and Altman (29). Spearman’s correlation coefficients were calculated for baseline-corrected peak/nadir values. Statistical analyses were performed using Graphpad Prism version 5.03 (Graphpad Software, La Jolla, CA, USA) and SPSS Statistics version 22 (IBM Corporation, Armonk, NY, USA). A *p*-value of less than 0.05 was considered statistically significant.

## Results

### Patient Characteristics

Patient characteristics are listed in Table 1. The majority of the patients were treated for peritoneal metastasis following a primary colon carcinoma (*n* = 18), and two patients were referred after peritoneal metastasis of a primary ovarian carcinoma. Mean procedural times were 249 [130–319] minutes for the CRS, 53 [42–74] minutes for the HIPEC, and 137 [108–170] minutes between the end of HIPEC and ICU admission. A complete macroscopic cytoreduction was achieved in all patients.

**Table 1:** Patient characteristics.

	All patients (*n* = 20)
Males	11 (55%)
Age (years)	61 [51–72]
Weight (kg)	78 [68–100]
Length (cm)	174 [168–180]
ECOG performance status	1 [1–1]
ASA classification	2 [2–3]
Length of surgery (min)	374 [244–446]
Peritoneal cancer index	9 [2–14]
Transfusion of blood products^a^	7 (35%)
Length of TPN administration (days)	7 [5–9]
Postoperative complications^b^	12 (60%)
Grade I	1 (5%)
Grade II	8 (40%)
Grade III	1 (5%)
Grade IV	2 (10%)
Postoperative infections	5 (25%)
ICU length of stay (days)	1.5 [1–2]
Hospital length of stay (days)	11 [8–12]
28-day survival	20 (100%)

*Data are presented as median [IQR] or number (percentage)*.

*Abbreviations: ECOG, Eastern Cooperative Oncology Group; ASA, American Society of Anesthesiologists; TPN, total parenteral nutrition; ICU, intensive care unit*.

*^a^Administration of erythrocytes or fresh frozen plasma within 24 h after start surgery*.

*^b^According the Clavien–Dindo classification, the administration of TPN is not taken into account as all patients received TPN*.

### Plasma DAMP and LDH Concentrations

**Figure 1:**
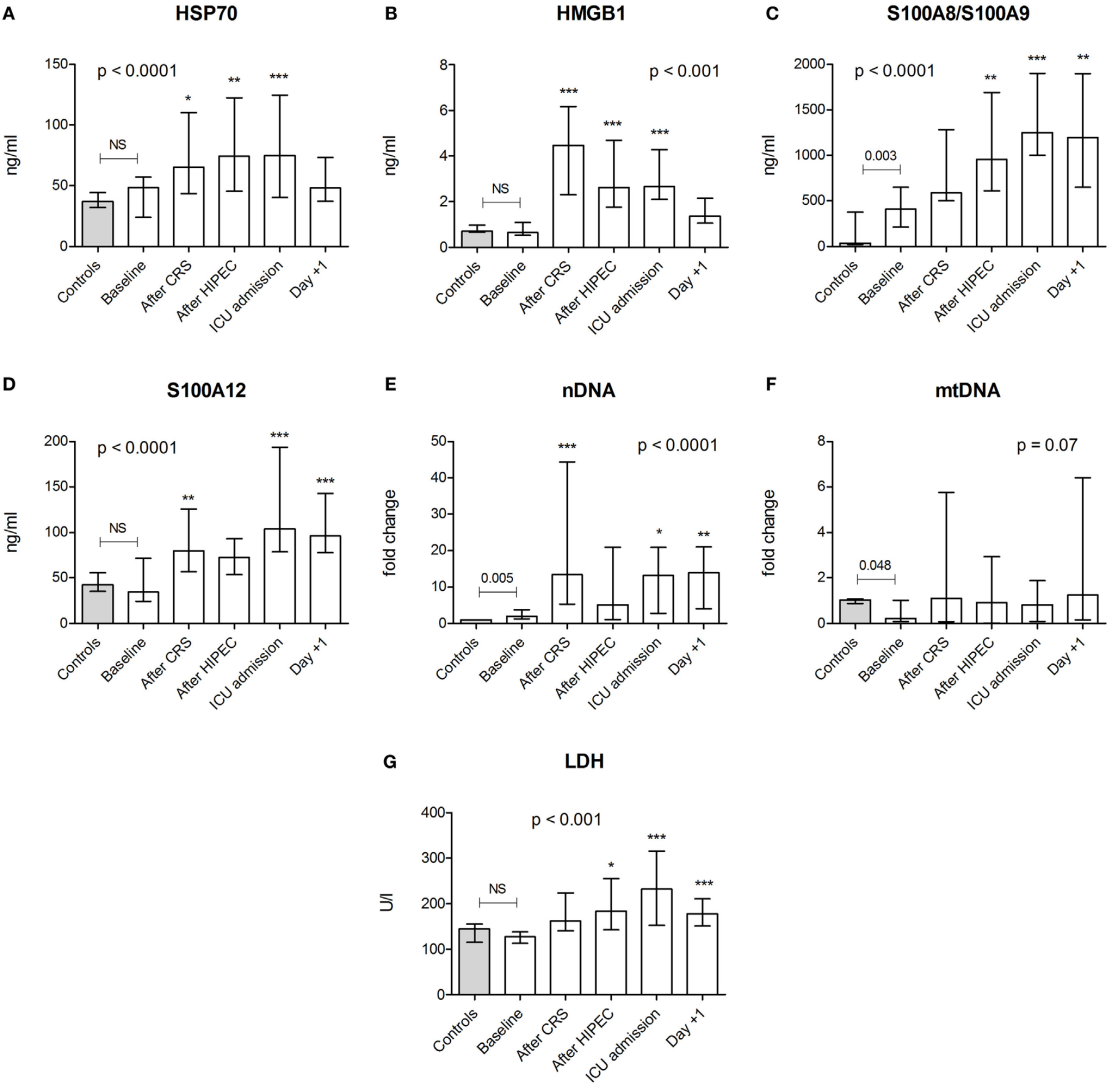
Plasma concentrations of danger-associated molecular patterns and lactate dehydrogenase (LDH). Plasma levels of **(A)** heat shock protein (HSP)70, **(B)** high mobility group box (HMGB)1, **(C)** S100A8/S100A9, **(D)** S100A12, **(E)** nuclear (n)DNA, **(F)** mitochondrial (mt)DNA, and **(G)** LDH in healthy controls (*n* = 30 for HSP70, S100A8/S100A9, and S100A12; *n* = 12 for HMGB1, LDH, nDNA, and mtDNA) and in patients (*n* = 20) at baseline and at various time points during and after the CRS-HIPEC procedure. nDNA and mtDNA levels are depicted as fold change compared to mean levels found in healthy controls. Data are presented as median + interquartile range. *p-*values indicating differences between controls and baseline patient data were calculated using Mann–Whitney *U*-tests. The *p-*values in each panel indicate differences within patient data over time and were calculated using Friedman tests with Dunn’s *post hoc* tests (*p < 0.05; **p < 0.01; ***p < 0.001 compared with baseline). Abbreviations: NS, not significant; CRS, cytoreductive surgery; HIPEC, hyperthermic intraperitoneal chemotherapy; ICU, intensive care unit; Day +1, first postoperative day.

**Figure 2:**
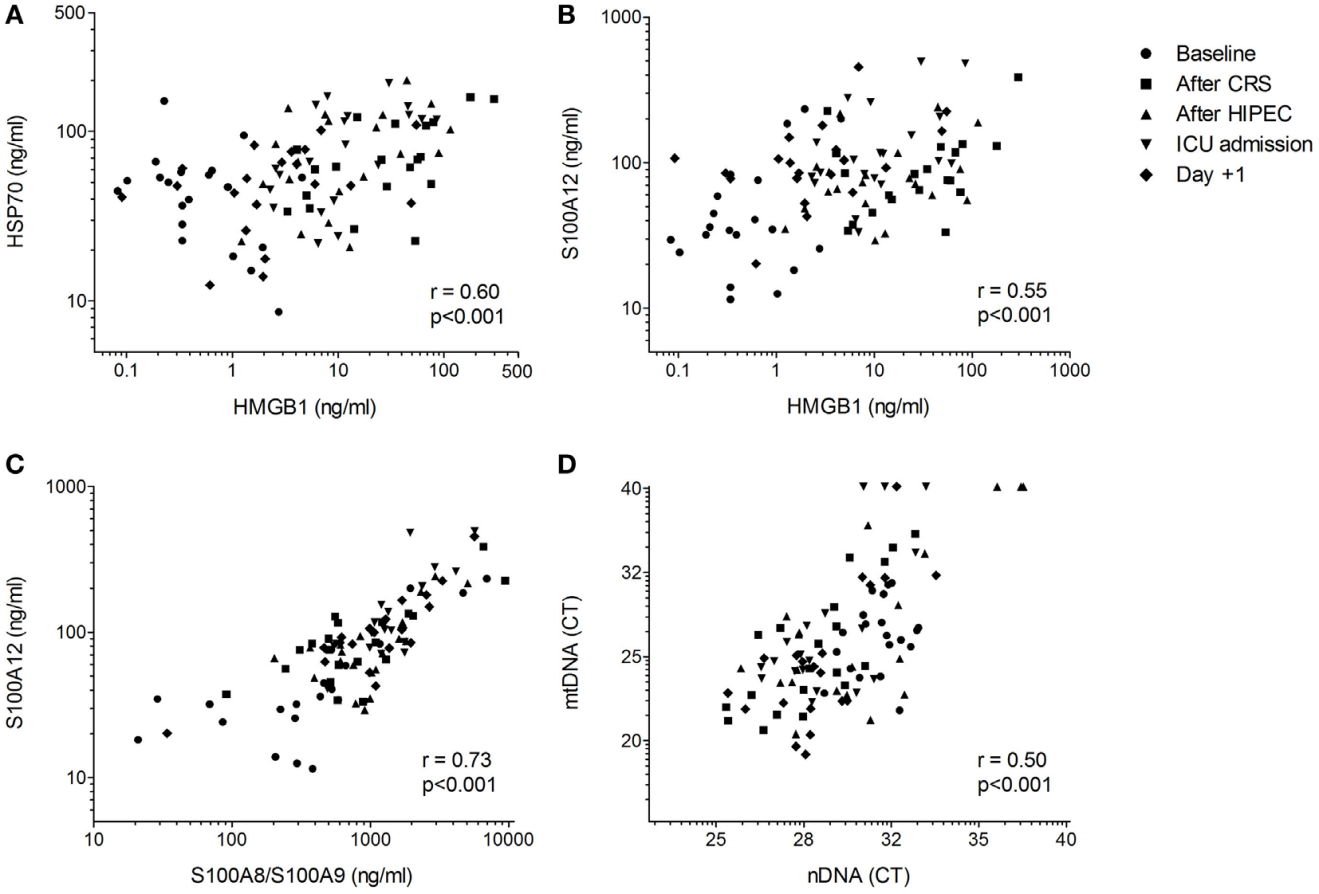
Correlations between danger-associated molecular patterns. Correlations between **(A)** high mobility group box (HMGB)1 and heat shock protein (HSP)70, **(B)** HMGB1 and S100A12, **(C)** S100A8/S100A9 and S100A12, and **(D)** nuclear (n)DNA and mitochondrial (mt)DNA in CRS-HIPEC patients (*n* = 20, five time points per patient). Correlations were determined using the method for repeated measurements according to Bland and Altman (29).

HSP70, HMGB1, S100A12, and LDH levels in HIPEC patients at baseline did not differ from values obtained in healthy controls. S100A8/S100A9 and nDNA levels at baseline were significantly higher, whereas mtDNA concentrations were significantly lower in patients (Figure 1). Plasma levels of HSP70, HMGB1, S100A8/S100A9, S100A12, nDNA, and LDH increased during the procedure, whereas mtDNA showed a trend toward increased levels. HMGB1 concentrations peaked directly after CRS, whereas levels of HSP70, S100A8/S100A9, S100A12, and LDH peaked at ICU admission. HSP70 and HMGB1 returned to baseline 1 day after the procedure, whereas S100A8/S100A9, S100A12, nDNA, and LDH concentrations remained elevated for the duration of the study. HMGB1 correlated with HSP70 and S100A12 (Figures 2A,B). Furthermore, both S100 proteins and both DNA’s were intercorrelated (Figures 2C,D).

### Plasma Cytokine Levels

**Figure 3:**
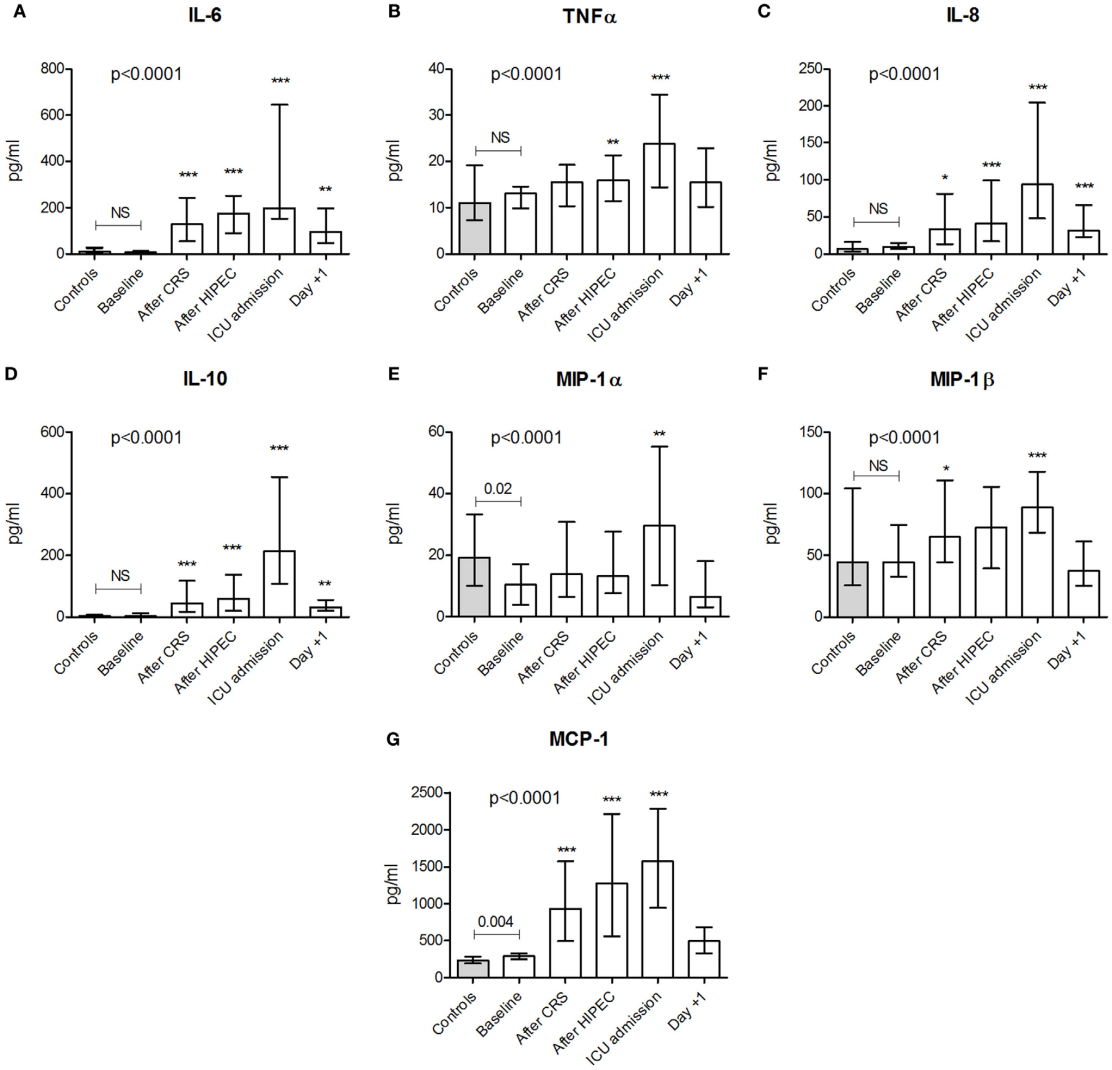
Plasma concentrations of cytokines. Plasma levels of **(A)** interleukin (IL)-6, **(B)** tumor necrosis factor α, **(C)** IL-8, **(D)** IL-10, **(E)** macrophage inflammatory protein (MIP)-1α, **(F)** MIP-1β, and **(G)** monocyte chemoattractant protein (MCP)-1 in healthy controls (*n* = 30) and in patients (*n* = 20) at baseline and at various time points during and after the CRS-HIPEC procedure. Data are presented as median + interquartile range. *p*-values indicating differences between controls and baseline patient data were calculated using Mann–Whitney *U*-tests. The *p*-values in each panel indicate differences within patient data over time and were calculated using Friedman tests with Dunn’s *post hoc* tests (*p < 0.05; **p < 0.01; ***p < 0.001 compared with baseline). Abbreviations: NS, not significant; CRS, cytoreductive surgery; HIPEC, hyperthermic intraperitoneal chemotherapy; ICU, intensive care unit; Day +1, first postoperative day.

Baseline plasma cytokine levels in the HIPEC patients were similar to those observed in healthy controls, except for slightly elevated values of MCP-1 and MIP-1α (Figure 3). Upon the CRS-HIPEC procedure, all cytokine levels significantly increased compared with baseline, with peak levels observed at ICU admission. Levels of TNFα, MIP-1α, MIP-1β, and MCP-1 returned to baseline 1 day after the procedure. At this time point, concentrations of IL-6, IL-8, and IL-10 were also decreased compared with their peak levels, but remained elevated compared to baseline. Except for mtDNA, all DAMPs and LDH correlated significantly with TNFα, the interleukins, and MCP-1, but only HSP70 and HMGB1 correlated with MIP-1α and MIP-1β (Table 2).

**Table 2:** Correlations between danger-associated molecular patterns (DAMPs), LDH, and plasma cytokines.

	HSP70	HMGB1	S100A8/S100A9	S100A12	nDNA	mtDNA	LDH
Tumor necrosis factor α	***r* = 0.31, *p* = 0.004**	***r* = 0.30, *p* = 0.007**	***r* = 0.29, *p* = 0.008**	***r* = 0.29, *p* = 0. 008**	*r* = 0.21, *p* = 0.06	*r* = −0.13, *p* = 0.261	***r* = 0.31, *p* = 0.005**
Interleukin (IL)-6	***r* = 0.60, *p* < 0.001**	***r* = 0.72, *p* < 0.001**	***r* = 0.56, *p* < 0.001**	***r* = 0.65, *p* < 0.001**	**r = 0.47, *p* < 0.001**	*r* = 0.09, *p* = 0.451	***r* = 0.62, *p* < 0.001**
IL-8	***r* = 0.57, *p* < 0.001**	***r* = 0.62, *p* < 0.001**	***r* = 0.64, *p* < 0.001**	***r* = 0.63, *p* < 0.001**	***r* = 0.48, *p* < 0.001**	*r* = −0.01, *p* = 0.994	***r* = 0.60, *p* < 0.001**
IL-10	***r* = 0.51, *p* < 0.001**	***r* = 0.72, *p* < 0.001**	***r* = 0.50, *p* < 0.001**	***r* = 0.62, *p* < 0.001**	***r* = 0.42, *p* < 0.001**	*r* = 0.04, *p* = 0.728	***r* = 0.51, *p* < 0.001**
Macrophage inflammatory protein (MIP)-1	***r* = 0.35, *p* = 0.001**	***r* = 0.39, *p* < 0.001**	*r* = 0.15, *p* = 0.191	*r* = 0.12, *p* = 0.284	*r* = 0.09, *p* = 0.476	*r* = −0.07, *p* = 0.541	*r* = 0.16, *p* = 0.16
MIP-1β	***r* = 0.45, *p* < 0.001**	***r* = 0.47, *p* < 0.001**	*r* = 0.18, *p* = 0.115	*r* = 0.15, *p* = 0.177	*r* = 0.01, *p* = 0.915	*r* = −0.24, *p* = 0.033	*r* = 0.13, *p* = 0.25
Monocyte chemoattractant protein (MCP)-1	***r* = 0.65, *p* < 0.001**	***r* = 0.73, *p* < 0.001**	***r* = 0.48, *p* < 0.001**	***r* = 0.48, *p* < 0.001**	***r* = 0.34, *p* = 0.002**	*r* = −0.06, *p* = 0.593	***r* = 0.50, *p* < 0.001**

*Correlations were determined using the method for repeated measurements according to Bland and Altman (29). Significant correlations are shown in bold*.

*Abbreviations: HSP70, heat shock protein 70; HMGB1, high mobility group box 1; nDNA, nuclear DNA; mtDNA, mitochondrial DNA; LDH, lactate dehydrogenase*.

### Immune Suppression

**Figure 4:**
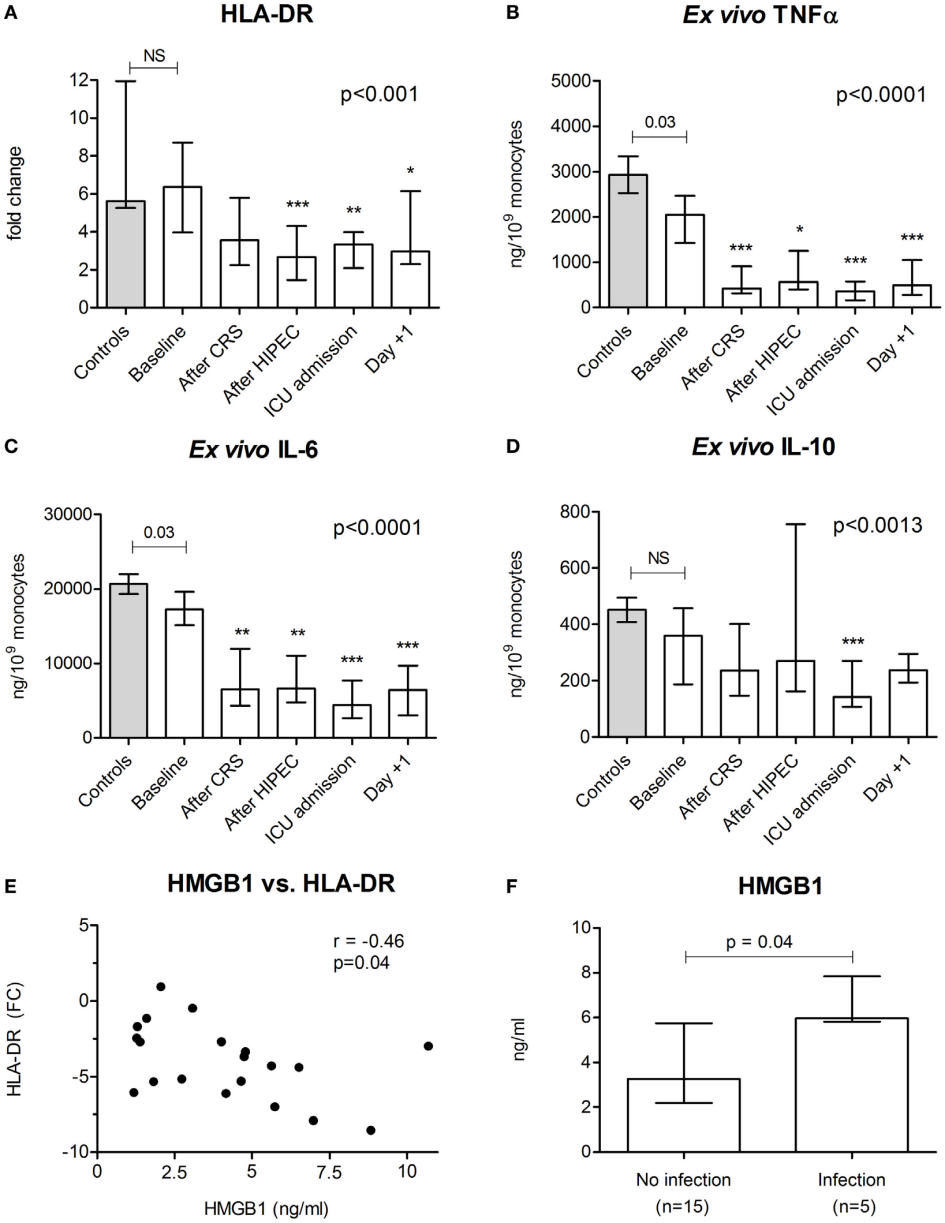
Markers of immune suppression and postoperative infections. **(A)** HLA-DR gene expression in leukocytes and production of **(B)** tumor necrosis factor α, **(C)** interleukin (IL)-6, and **(D)** IL-10 by leukocytes *ex vivo* stimulated with lipopolysaccharide in healthy controls (*n* = 12) and in patients (*n* = 20) at baseline and at various time points during and after the CRS-HIPEC procedure. **(E)** Correlation between the maximum increase in high mobility group box (HMGB)1 and the maximum decrease in HLA-DR expression. **(F)** Peak HMGB1 plasma levels between patients who went on to develop a postoperative infection and patients who did not. HLA-DR levels are depicted as fold change compared to reference gene peptidylpropylisomerase B. Data in panels **(A–D)** are depicted as median + interquartile range. The *p*-values in panels **(A–D)** indicating differences between controls and baseline patient in data were calculated using Mann–Whitney *U*-tests. The *p*-values in panels **(A–D)** indicate differences within patient data over time and were calculated using Friedman tests with Dunn’s *post hoc* tests (*p < 0.05; **p < 0.01; ***p < 0.001 compared with baseline). Spearman correlation was used in panel **(E)**. The *p*-value in panel **(F)** was calculated using the Mann–Whitney *U*-test. Abbreviations: NS, not significant; CRS, cytoreductive surgery; HIPEC, hyperthermic intraperitoneal chemotherapy; ICU, intensive care unit; Day +1, first postoperative day.

At baseline, leukocytic HLA-DR expression was similar between HIPEC patients and healthy controls (Figure 4A). During the procedure, HLA-DR expression decreased with the lowest expression observed directly after the HIPEC procedure. Baseline production of TNFα and IL-6 by leukocytes *ex vivo* stimulated with LPS was lower in patients compared with healthy controls, whereas *ex vivo* IL-10 production was not significantly different (Figures 4B–D). During and after the CRS-HIPEC procedure, *ex vivo* production capacity was impaired for all cytokines. This effect was most pronounced for the pro-inflammatory cytokines TNFα and IL-6. The increase in HMGB1 levels (peak-baseline) correlated with the decrease in HLA-DR expression (*r* = −0.46, *p* = 0.04, Figure 4E). No significant correlations between other DAMPs and HLA-DR expression were observed.

### Postoperative Infections

Five (25%) HIPEC patients developed one or more severe postoperative infections during the follow up period, with a median time of 7 [6–9] days between surgery and development of the infection. Two patients developed two infections. Primary foci were central line-related bloodstream infection (*n* = 3), pneumonia (*n* = 2), and abdominal infection (*n* = 2). All three patients developing a central line received TPV at the time of infection. A diaphragmatic resection was performed in three cases. In one of these patients, the thoracic cavity was also perfused with oxaliplatin, which was complicated by a pneumonia. Peak HMGB1 concentrations were significantly higher in patients who went on to develop an infection (*p* = 0.04, Figure 4F). No such relationships were observed for other DAMPs or cytokines.

## Discussion

In this study, we demonstrate that CRS-HIPEC results in DAMP release, a systemic inflammatory response, and development of an immune-suppressed phenotype, the latter exemplified by a decrease in leukocytic HLA-DR expression and diminished *ex vivo* pro-inflammatory cytokine production. This immune suppressive phenotype was already observed immediately following CRS, and became more pronounced following HIPEC. Despite normalization of inflammatory cytokines in the circulation, immune suppression persisted 1 day after surgery. Furthermore, HMGB1 levels were related to the occurrence of postoperative infections in CRS-HIPEC patients.

Our data show that the CRS-HIPEC procedure results in the release of DAMPs and LDH, suggesting noticeable cellular damage, (unscheduled) cell death, and/or cellular stress. This observation is in accordance with previous studies of our group in cohorts of trauma, sepsis, cardiac arrest, and chemotherapy-treated leukemia patients (13, 19, 30, 31). It is well known that virtually all DAMPs are released as a result of cellular rupture and/or decay (23, 32, 33). Next to this passive release mechanism, several forms of active DAMP release have been described. For instance, several proteins of the HSP70 family, representing a variety of chaperone proteins residing in the cytoplasm, nucleus, extracellular exosomes, and on the cellular membrane (34), can be released upon hyperthermic stress (25). S100 proteins, implicated in numerous regulatory functions, are released into the circulation upon metabolic or oxidative stress (23). Moreover, the cytokine-like factor HMGB1 is highly abundant in colorectal tumor cells (35), and can be released into the circulation by activated immune cells, and by passive leakage from necrotic (tumor) cells (36). nDNA and mtDNA, present in all nucleated cells, are thought to be largely released upon cell rupture/decay (24), although it has been suggested that these can also be actively secreted (37). The latter is supported by recent data showing increased levels of nDNA in a relatively mild model of systemic inflammation in human *in vivo* (31).

In this study, patients underwent a double hit of surgical trauma (CRS) followed by hyperthermic chemotherapy (HIPEC). We found increased levels of those DAMPs reflecting cellular decay and of LDH (a nonspecific marker of unscheduled cell death) directly after CRS, and relatively strong correlations between the various DAMPs measured. This suggests that mechanical rupture of cells, leading to nonspecific release of various DAMPs, likely plays a more important role in the increased DAMP levels encountered than the HIPEC procedure. The rapid release of HMGB1 after mechanical trauma is in accordance with previous work (38–40). Perhaps not surprisingly, plasma levels of HMGB1 were markedly higher in patients directly after severe mechanical trauma (38) and blunted chest trauma (39) compared to the levels found in CRS-HIPEC patients in this study, whereas similar levels were observed in septic patients (40). Moreover, elevated levels of HMGB1 have also been demonstrated in severe burn trauma (41). Although oxaliplatin used in the HIPEC procedure exerts cytotoxic effects which are mainly caused by inflicting DNA damage (42), and several studies demonstrate release of DAMPs from apoptotic chemotherapy-treated cancer cells (43, 44), it is unlikely that this contributes to the early DAMP release in our patient cohort, as these effects are thought to manifest in a later stage (44). Nevertheless, it is conceivable that chemotherapy-mediated apoptosis could contribute to prolonged DAMP release in these patients. Interestingly, similar to what was observed in trauma and chemotherapy-treated patients (13, 19), but unlike findings in patients with septic shock or cardiac arrest (30, 31), mtDNA levels did not significantly increase in our patient cohort, and no correlations with other DAMPs were present. This suggests that other release or clearance mechanisms exist for this DAMP, as has been proposed before (30).

Our data reveal that CRS-HIPEC patients display higher levels of S100A8/S100A9 at baseline compared to healthy controls. This is likely a result of colorectal metastatic disease, as S100A8/S100A9 is an important mediator in cancer pathology which induces growth and stimulates metastasis (45). In accordance with our data, circulating levels of nDNA were also reported to be higher in patients with colorectal cancer compared to healthy controls (46), and interestingly, nDNA has been proposed as a diagnostic and prognostic predictor of colorectal cancer (47).

The suspected interrelation between DAMPs and the inflammatory response is supported by our observation that all plasma cytokines increased during and following CRS-HIPEC, and all DAMPs except for mtDNA correlated well with circulating cytokine levels. The absence of a significant increase in mtDNA levels likely explains the lack of correlation for this DAMP. As such, our data are in line with literature showing that various DAMPs can elicit immune responses *via* ligation of pattern recognition receptors. HSP70 is recognized by toll-like receptor (TLR)2 and TLR4, ligation of both elicits the release of inflammatory cytokines (48). HMGB1 triggers and sustains inflammation and recruits leukocytes by binding to TLRs (49), the receptor for advanced glycation endproducts (RAGE), and T-cell immunoglobulin and mucin-domain containing (TIM)-3 (50). In addition, S100A8/S100A9 binds to a plethora of receptors, including TLRs and RAGE, whereas S100A12 specifically binds to RAGE (36, 51). Via nuclear factor-kappa B activation, both S100 proteins promote production of inflammatory cytokines (51). nDNA, next to serving as a general indicator of cellular damage, may induce inflammatory responses through ligation of retinoid acid-inducible gene-1 and DNA-dependent activator of interferon regulatory factors (52).

Patients clearly developed immune suppression during and after the CRS-HIPEC procedure, exemplified by a decrease in HLA-DR expression, the extent of which was comparable to that previously observed in trauma patients (13), and reduced production of especially pro-inflammatory cytokines by *ex vivo* stimulated leukocytes. Several studies have demonstrated that DAMPs can cause immune suppression or the related phenomenon endotoxin tolerance. This has specifically been described for HMGB1 (53, 54), HSP70 (55, 56), and S100A8/S100A9 (20, 57, 58). In addition to DAMP-induced immune dysfunction, recent literature shows that immune function can be further suppressed by anesthetic drugs, such as ketamine and propofol (59), which were used in our study population. Therefore, we cannot rule out effects of the anesthetic regime on immune function in this study.

The decrease in HLA-DR expression is a widely known and studied hallmark of immune suppression, especially in the sepsis field (16). Interestingly, the increase in plasma HMGB1 levels was associated with the decrease in HLA-DR expression and, importantly, with the development of postoperative infections, suggestive of a dominant role for this DAMP. These data are in line with previous work in severe blunt chest trauma patients, where HMGB1 concentrations were associated with an increased risk for sepsis (39). Moreover, in septic shock patients, HMGB1 levels were significantly lower in the group of patients surviving the sepsis episode (40), and peak levels of HMGB1 following gastrointestinal surgery correlated with the duration of the systemic inflammatory response as well as with postoperative pulmonary dysfunction (60).

This study has several shortcomings. First and foremost, due to the observational nature of this study, only associations and not causative effect can be deduced. Second, our small sample size in combination with a relatively large quantity of measured variables does not allow an exploratory factor analysis to identify components that discriminate between groups. Third, a larger cohort is required to confirm the observed relationship between HMGB1 and postoperative infections, and to determine its predictive value. Fourth, the follow up period of our study was limited to only 1 day. We chose to use such a short follow up period because we first wanted to establish whether CRS-HIPEC actually resulted in DAMP release and immunological sequelae. As such, future studies need to reveal the duration of immune suppression and further unravel its relation with postoperative infections. Finally, although we hypothesize that CRS is most important, we cannot draw definitive conclusions on whether CRS or HIPEC is the dominant procedure causing DAMP release and immune suppression, because both procedures follow each other within a very short time frame. A study including patients, where CRS and HIPEC are performed separately with a reasonable time interval could shed light on this issue. However, this approach is rarely used in clinical practice.

## Conclusion

This study demonstrates early DAMP release and immune suppression in patients undergoing CRS-HIPEC. Furthermore, our findings of immune suppression at the biochemical level are supported by the high incidence of serious postoperative infections (25%) in our cohort, which is in line with previous data (8, 10–12). Interestingly, plasma levels of HMGB1 were related to the occurrence of postoperative infections. These data emphasize the need to further unravel the mechanisms behind immune dysfunction following CRS-HIPEC and similar procedures. If a causative relationship between DAMP-induced immune suppression and postoperative infections can be established in larger cohorts, it could pave the way for future research in the field of immune dysfunction following these procedures. Early recognition could direct adjunctive immunotherapy to those patients who could actually benefit. Similar advances are made in the sepsis field, were immunostimulatory agents are being proposed for the reversal of immune suppression (61–63). Moreover, therapies targeting HMGB1, but also other DAMPs, might be adopted from the oncology and sepsis fields (64–66), and applied for use in surgery and/or chemotherapy-induced immune suppression.

## Ethics Statement

The local ethics committee that reviewed the protocol and approved this study is the CMO Arnhem-Nijmegen (Reference no. 2017-3607). Control samples were obtained from healthy volunteers participating in two other studies. These studies were also approved by the CMO Arnhem-Nijmegen (Reference no’s. 2012-455 and 2016-2550). All patients and all healthy volunteers provided written informed consent. All study procedures were conducted in accordance with the declaration of Helsinki, including current revisions, and Good Clinical Practice guidelines.

## Author Contributions

GL, HC, PP, and MK designed the study. GL, HC, and AH included the patients, obtained, and processed the samples. JG, JR, and TV performed the laboratory analyses. GL and HC performed the statistical analyses and wrote the manuscript. TV, GS, PP, and MK critically reviewed the manuscript and supervised the research. All authors reviewed the manuscript.

## Conflict of Interest Statement

The authors declare that the research was conducted in the absence of any commercial or financial relationships that could be construed as a potential conflict of interest.
